# Pituitary stalk thickening in pediatric patients: an underrecognized diagnosis?

**DOI:** 10.20945/2359-4292-2023-0396

**Published:** 2024-08-09

**Authors:** Diego Zepeda, Francisco Javier Guarda, Cecilia Okuma, María Isabel Hernández

**Affiliations:** 1 Universidad de Chile Facultad de Medicina Instituto de Investigaciones Materno Infantil Santiago Chile Instituto de Investigaciones Materno Infantil (IDIMI), Facultad de Medicina, Universidad de Chile, Santiago, Chile; 2 Pontificia Universidad Católica de Chile Facultad de Medicina Departamento de Endocrinología y Centro de Endocrinología Traslacional Santiago Chile Departamento de Endocrinología y Centro de Endocrinología Traslacional (CETREN), Facultad de Medicina, Pontificia Universidad Católica de Chile, Santiago, Chile; 3 Programa de Cirugía Endoscópica Endonasal de Base de Cráneo y Tumores Pituitarios Red de Salud UC-CHRISTUS Santiago Chile Programa de Cirugía Endoscópica Endonasal de Base de Cráneo y Tumores Pituitarios, Red de Salud UC-CHRISTUS, Santiago, Chile; 4 Instituto de Neurocirugía Asenjo Santiago Chile Instituto de Neurocirugía Asenjo, Santiago, Chile; 5 Universidad de Chile Facultad de Medicina Departamento de Ciencias Neurológicas Santiago Chile Departamento de Ciencias Neurológicas, Facultad de Medicina, Universidad de Chile, Santiago, Chile

**Keywords:** Pituitary stalk thickening, pituitary hormones, child, pituitary stalk, germinoma

## Abstract

**Objective:**

Pituitary stalk thickening (PST) is a rare condition in pediatric patients. Data on PST in Latin American pediatric populations are scarce. The aim of this study was to characterize a comprehensive cohort of pediatric patients diagnosed with PST in Chile between 2020 and 2022.

**Subjects and methods:**

Retrospective review of medical records from 2020 to 2022 of patients under 18 years old diagnosed with PST, defined as a pituitary stalk width ≥ 3 mm at the pituitary insertion and/or ≥ 4 mm at the optic chiasm. A literature review was also performed to compare the identified cases with previously published ones.

**Results:**

Nine patients with PST were identified. Their mean age at diagnosis was 10.36 years (range 2.4-17 years). The patients’ main manifestations were polydipsia and polyuria (100%) and poor growth (77.8%). Eight patients had germ cell tumors, while one patient had Langerhans cell histiocytosis. At the time of diagnosis, all patients had arginine vasopressin (AVP) deficiency, along with a deficiency in at least one anterior pituitary hormone. Germ cell tumor markers were negative in all patients. A biopsy-confirmed diagnosis was obtained in all cases. Four patients required a second biopsy. The frequency of PST due to germ cell tumors was four patients/year during the study period, which is twice the expected frequency in Chile.

**Conclusion:**

This study, characterizing the largest cohort of pediatric patients with PST in Latin America, found germ cell tumors to be the main etiology of this condition. It is important to focus diagnostic procedures on obtaining a correct diagnosis and promptly initiating appropriate treatment in patients with PST. Regional cooperation is essential for gathering data from larger cohorts to enhance our understanding of pediatric PST and improve patient outcomes.

## INTRODUCTION

Pituitary stalk thickening (PST) is rare in pediatric patients, with only a few published studies focused on this age group. Consequently, the definition of PST in the pediatric population is controversial and difficult to establish. The advances in imaging techniques and the use of magnetic resonance imaging (MRI) have been fundamental in characterizing the pituitary stalk size and shape properly and providing a better understanding of the disorders that affect this anatomical region (1).

In adults, the transverse diameters considered normal for the pituitary stalk are 1.91 ± 0.4 mm at the pituitary insertion and 3.25 ± 0.56 mm at the optic chiasm (2). In this age group, PST is defined by a pituitary stalk width > 3 mm (3). Among children, the definition of PST has been controversial due to scarce data and lack of neuroimaging obtained from healthy pediatric patients. Recently, a consensus guideline by UK experts defined PST in children as a pituitary stalk width ≥ 3 mm at pituitary insertion and/or ≥ 4 mm at the optic chiasm (4).

Patients with PST may have neuroendocrine and/or visual impairment. The most frequent clinical manifestations of PST are anterior pituitary dysfunction, arginine vasopressin (AVP) deficiency, visual field defects, and signs of increased intracranial pressure. In terms of pituitary dysfunction, the most common clinical presentation is polyuria and polydipsia (5) due to AVP deficiency. Deficiency of anterior pituitary hormones occurs in half of the patients (2,6).

The causes of PST vary widely, covering a broad range of conditions, including neoplastic, congenital, inflammatory/infectious, and autoimmune diseases. In pediatric patients, the most common causes are neoplasms, followed by congenital lesions. Among the neoplasms, the most frequent are germ cell tumors, followed by Langerhans cell histiocytosis (7,8). The congenital lesions most frequently described are pituitary hypoplasia and Rathke's cleft cysts (7,8). Inflammatory/infectious and autoimmune diseases causing PST in the pediatric population are extremely rare (2).

The etiological diagnosis of PST is challenging. After a detailed history and an extensive clinical evaluation, the first-line investigations in patients with PST include general laboratory workup, serum tumor markers, anterior and posterior pituitary function, visual assessment, and imaging studies for identification of lesions compatible with Langerhans cell histiocytosis. If the etiology remains unidentified, a patient with a PST < 6.5 mm and without AVP deficiency, worsening of anterior pituitary deficiency, or visual impairment may be monitored with clinical evaluations and MRI scans every 6 months for the first 2 years, then annually thereafter (9). If the patient exhibits any of these manifestations, second-line investigation is required, including cerebrospinal fluid (CSF) measurement of tumor markers and whole-body imaging, particularly in those patients with progressive disease. Pituitary stalk biopsy should be considered for patients whose second-line investigation yields negative results (2).

An MRI obtained at the time of PST diagnosis can provide additional information. Some features, such as pituitary hypoplasia and absence of the posterior pituitary bright spot (as observed on T1-weighted imaging [T1WI]) or midline brain defects direct the diagnosis toward non-neoplastic etiologies (10).

Recently in Chile, we have observed an unexpected rise in the number of pediatric cases of PST. In the present study, we conducted a retrospective analysis of the clinical, biochemical, radiological, and histological characteristics of pediatric patients diagnosed with PST between 2020 and 2022 in Chile. This is the first comprehensive analysis of a pediatric PST cohort in Latin America.

## MATERIALS AND METHODS

The study protocol was approved by our institutional review board and local ethics committee.

We conducted a retrospective review of clinical, radiological, and histological data from nine patients with PST diagnosed between 2020 and 2022. The data were collected from electronic medical records at the *Instituto de Neurocirugía, Asenjo* (INCA) in Santiago, Chile, and from referrals from other centers. We also actively searched for patients with PST by consulting neurosurgeons, oncologists, and pediatric endocrinologists throughout the country. Due to national policies, all pediatric patients with cancer are registered in a centralized database of Chile's National Childhood Antineoplastic Drug Program (PINDA). Further, we compared our findings with the expected data for the same period in Chile.

The inclusion criteria were age < 18 years and diagnosis of PST, as defined by the UK consensus (*i.e.*, pituitary stalk ≥ 3 mm at the pituitary insertion and/or ≥ 4 mm at the optic chiasm).

The information collected included the patients’ weight, height, and body mass index obtained at presentation, pubertal development (Tanner stage), as defined by a pediatric endocrinologist, manifestations of anterior and posterior pituitary hormone deficiencies, and neuro-ophthalmologic symptoms. Other symptoms described in the patients’ medical records were also included.

The anterior pituitary function was assessed at the first clinical visit by measuring levels of thyroid-stimulating hormone (TSH), thyroid hormones, adrenocorticotropic hormone (ACTH), cortisol, luteinizing hormone (LH), follicle-stimulating hormone (FSH), testosterone or estradiol, and prolactin. Serum insulin-like growth factor 1 (IGF-1) levels and, when required, growth hormone (GH) stimulation test were also obtained. A diagnosis of GH deficiency was established in patients with clinical manifestations associated with low IGF-1 levels, *i.e.*, stature < -2 standard deviation scores (SDS) and/or growth velocity below the 25th percentile. A GH stimulation test with clonidine was also obtained in patients with suspected GH deficiency (two patients underwent this evaluation). The diagnosis of GH deficiency was established with a GH peak < 7 µg/L. Hypothalamus-pituitary-adrenal (HPA) axis insufficiency was diagnosed in the presence of serum cortisol level at 8:00 AM < 80 nmol/L or peak cortisol level < 497 nmol/L after low-dose (1 µg) ACTH stimulation test. A diagnosis of central hypothyroidism was established in patients with a low free T4 level associated with an inappropriately normal or low TSH level. Dysfunction of the hypothalamus-pituitary-gonadal (HPG) axis was diagnosed in patients with pubertal arrest, amenorrhea with low gonadotropin levels, and low estradiol or testosterone levels. Hyperprolactinemia was defined as prolactin levels above the normal reference range.

The posterior pituitary function was evaluated through the calculation of serum osmolality (Osmp), estimated from serum levels of sodium, glucose, and urea, and urine osmolality (Osmu), estimated from urine density. A diagnosis of AVP deficiency was established in patients with polydipsia and polyuria associated with Osmp > 300 mOsm/kg and OsmU < 300 mOsm/kg.

Markers of germ cell tumors (*i.e.*, alpha-fetoprotein [AFP] and human chorionic gonadotropin [hCG]) were measured both in serum and cerebrospinal fluid (CSF) in all patients.

All patients underwent neuro-ophthalmologic evaluation. Visual field was assessed using a Goldmann campimetry.

Imaging of the pituitary region was obtained using a 3-Tesla (Siemens Skyra) or 1.5-Tesla (Philips Medical Systems) MRI scanner. The images were read by an expert neuroradiologist, who described the anatomy of the pituitary region. The pituitary stalk was measured at the levels of the pituitary insertion and optic chiasm, and the maximum diameter at each of these levels was defined. Measurement of the pituitary was also obtained, and the presence or absence of the posterior pituitary bright spot was noted. Additionally, the occurrence of midline brain defects or optic chiasm involvement was described.

The PST etiology was established by biopsy in all patients. When a patient underwent more than one biopsy, the results of all biopsies were analyzed.

We also conducted a literature review to compare and contextualize our results with those from previously published studies.

## RESULTS

Nine patients (seven girls and two boys) with PST were identified during the study period. The mean age at diagnosis was 10.36 years (range 2.4-17 years). The main reasons for the first visit were short stature, as well as polydipsia and polyuria. Other symptoms included headache, pubertal arrest, visual disturbances, primary amenorrhea, and jaundice.

At diagnosis, all patients had polydipsia and polyuria, while 77.8% had short stature or poor growth. Half of the patients had neuro-ophthalmologic symptoms. Three patients presented with headache and one with primary amenorrhea. A patient, who had Langerhans cell histiocytosis, exhibited features of short stature and cholestatic hepatitis at diagnosis, but no skin or skeletal lesions (Table 1).

**Table 1:** Symptoms at diagnosis in patients with pituitary stalk thickening

Symptoms	Number of patients (%)
Short stature and/or poor growth	7 (77.8)
Symptoms of hypothyroidism	3 (33.3)
Asthenia	2 (22.2)
Polydipsia and polyuria	9 (100)
Amenorrhea or delayed puberty	3 (33.3)
Neuro-ophthalmologic symptoms	5 (55.5)
Headache	3 (33.3)

On hormone assessment, all patients had both AVP and GH deficiencies. Seven patients had central hypothyroidism, five had central adrenal insufficiency, three had hypogonadotropic hypogonadism, and six had hyperprolactinemia (Table 2). Markers of germ cell tumors were negative in all patients. Five patients had visual field defects.

**Table 2:** Endocrine abnormalities at diagnosis in patients with pituitary stalk thickening

Endocrine abnormality	Number of patients (%)
Arginine vasopressin deficiency	9 (100)
Growth hormone deficiency	9 (100)
Hypothalamic-pituitary-adrenal axis	5 (55.5)
Hypothalamic-pituitary-gonadal axis	3 (33.3)
Hyperprolactinemia	6 (66.7)
Hypothalamic-pituitary-thyroid axis	7 (77.8)

**Figure 1:**
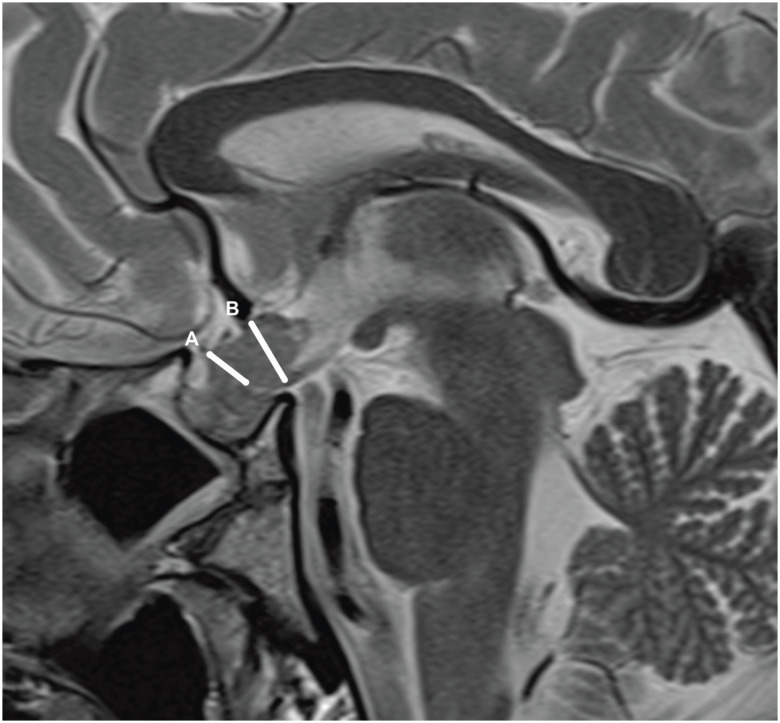
Magnetic resonance imaging of a patient with pituitary stalk thickening at the (A) pituitary insertion and (B) optic chiasm.

At the initial MRI, the median maximum diameter of the patients’ pituitary stalk was 8.1 mm (range 4.6-13 mm), and six patients had optic chiasm abnormalities due to compression. None of the patients had midline brain defects. Remarkably, the posterior pituitary bright spot was absent on T1WI in all patients (Figure 1).

The diagnosis of PST was determined by biopsy in all patients. In one patient, the diagnosis was established by liver biopsy during an investigation of a hepatic lesion, which revealed to be Langerhans cell histiocytosis on histology. The other eight patients underwent pituitary stalk biopsy.

The definitive diagnosis of the PST cause required a second biopsy in four patients – three were diagnosed with hypophysitis in the first biopsy and, due to PST progression, required a second biopsy, and one required two liver biopsies to establish the diagnosis of Langerhans cell histiocytosis.

The final etiology of PST was pure germ cell tumor in eight patients and Langerhans cell histiocytosis in one patient.

Table 3 summarizes the clinical and radiological characteristics of all patients.

**Table 3:** Description of patients with pituitary stalk thickening

Patient number	Age at diagnosis (years)	Sex	D_max_ (mm)	Endocrine abnormalities	Neuro-ophthalmologic symptoms	Tumor markers*	Diagnosis
1	7.1	F	4.6	AVD, GHD, HPT, HPA, HPRL	Present	Negative	Pure GCT
2	13.9	F	7.64	AVD, GHD, HPT, HPA, HPG, HPRL	Present	Negative	Pure GCT
3	7.3	M	5.0	AVD, GHD, HPA, HPRL	Absent	Negative	Pure GCT
4	15.6	F	8.46	AVD, GHD, HPT, HPA, HPG, HPRL	Present	Negative	Pure GCT
5	8.1	F	13.0	AVD, GHD, HPT, HPRL	Present	Negative	Pure GCT
6	17	F	12.5	AVD, GHD, HPT, HPA, HPG	Present	Negative	Pure GCT
7	10.3	F	8.1	AVD, GHD, HPT	Absent	Negative	Pure GCT
8	2.4	M	10.0	AVD, GHD, HPT	Absent	Negative	Langerhans cell histiocytosis
9	11.5	F	5.16	AVD, GHD, HPRL	Absent	Negative	Pure GCT

* Human chorionic gonadotropin (hCG) and alpha-fetoprotein (AFP), both measured in serum and cerebrospinal fluid.

Abbreviations: AVD, arginine vasopressin deficiency; Dmax, maximum diameter of the pituitary stalk; F, female; GCT, germ cell tumor; GHD, growth hormone deficiency; HPA, hypothalamic-pituitary-adrenal axis; HPG, hypothalamic-pituitary-gonadal axis; HPRL, hyperprolactinemia; HPT, hypothalamic-pituitary-thyroid axis; M, male.

## DISCUSSION

The present study described the cases of nine pediatric patients with PST who underwent a thorough evaluation of hormone profile, markers of germ cell tumors, neuroradiologic characteristics, and pathology results between 2020 and 2022 in Chile. In eight of these patients, the PST was caused by germ cell tumors. In Chile, the management of neoplastic diseases in children is guaranteed by social security, which ensures access to diagnosis and treatment and mandates notification. The patients in the study were assessed and treated under Chile's National Childhood Antineoplastic Drug Program (PINDA). Consequently, the maintenance of the medical records is mandatory for these patients, ensuring the accuracy of the data.

In the present cohort, the occurrence of PST was more frequent in girls than boys. Insufficient data are available to determine differences between sexes in patients with PST caused by germ cell tumors. In a case series of 11 patients with germ cell tumors with isolated pituitary stalk involvement identified over 6 years (2015-2021), Chen and cols. found no significant differences between sexes (5). Notably, germ cell tumors are reported to be more frequent in the male than female sex, with a ratio of about 4 to 1 (11).

All patients in the present study were diagnosed with AVP deficiency and at least one anterior pituitary hormone deficiency. In other similar studies, up to two-thirds of the patients with PST had AVP deficiency, and half had one or more deficiencies in anterior pituitary hormones (7,8,12).

The finding of nine patients with PST during the 2-year period of the present study was unexpectedly high compared with most literature reports, especially given the small size of the population under 17 years of age in Chile (approximately 4.2 million) (13). It is important to note that the study period overlapped with the COVID-19 pandemic, which may have delayed patient referrals, a trend also observed with other medical conditions during this time (14). The COVID-19 pandemic has considerably impacted health care systems, with studies indicating a rise in delayed referrals of pediatric patients with brain tumors during this period (15). In our cohort, all patients had a neoplastic cause for PST, and the most frequent neoplasia was pure germ cell tumors. Our finding of neoplasms being the most frequent cause of PST is consistent with the findings of previous studies (7).

In a case series of 65 patients with infundibular lesions identified from 1995 to 2003, Hamilton and cols. reported that 21 of these cases were in pediatric patients (8). Specifically, the authors found 13 congenital lesions (all in patients with pituitary hypoplasia) and eight tumors in these pediatric patients. Of the neoplastic causes, four were Langerhans cell histiocytosis, two were germ cell tumors, one was a metastatic glioblastoma multiforme, and one was a primitive neuroectodermal tumor.

Table 4 summarizes the published data on PST in pediatric patients. A 2013 study conducted at Mayo Clinic identified 152 pituitary stalk lesions diagnosed over a 20-year period (12). The causes, identified in 61% of the patients, were mainly attributed to neoplastic lesions (32%), inflammatory lesions (20%), and congenital anomalies (9%). Only 11% of the patients were younger than 21 years. Langerhans cell histiocytosis was classified as an inflammatory lesion in the Mayo Clinic study, rendering the neoplastic causes even more prevalent. On MRI, the most frequent finding of infundibulo-hypophyseal involvement among the cohort was a uniform enhancement in V-shape and round/diamond configuration (12). Zhou and cols. (7) described 321 cases of PST diagnosed from 2014 to 2017, including 91 in pediatric patients (28.3%). Among these pediatric patients, the mean age at PST diagnosis was 11 years. The most common causes of PST were neoplasms, and the most frequent tumors in pediatric patients were germ cell tumors (67%), followed by Langerhans cell histiocytosis (19%), and craniopharyngioma (7%). The authors found 16 patients with congenital lesions, all of which were identified as Rathke's cleft cysts. Yoon and cols. (16) described 76 pediatric patients with pituitary stalk lesions diagnosed between 2000 and 2013. Among them, 68.4% were neoplasms and 21.1% were congenital lesions. Of the neoplastic lesions, intracranial germ cell tumor was the most common etiology (40.4%), followed by Langerhans cell histiocytosis (28.8%). More recently, Moszczyńska and cols. reported a cohort of 23 patients with PST diagnosed between 1990 and 2020 in Poland (17). The median age at diagnosis in their study was 9.68 years. The most common cause of PST was germ cell tumors (73.9%), followed by Langerhans cell histiocytosis. Polyuria and polydipsia were present in 82.6% of the patients (17). These findings combined indicate that neoplasms are the most frequent cause of PST in pediatric patients. Therefore, a prompt diagnosis is fundamental for improved outcomes.

**Table 4:** Review of published data on pituitary stalk thickening in pediatric patients (references 7, 8, 12, 16, 17, 24).

Reference	Pediatric patients number (%)	Sex (% females)	GCT (%)	LCH (%)	Others (%)
Turcu and cols. (2013)	17 (11%)	60	6.5	7.60	85.9
Zhou and cols. (2019)	91 (28.3%)	55.5	29.9	20.4	49.7
Hamilton and cols. (2007)	21 (32.3%)	ND	6.2	6.2	87.6
Yoon and cols. (2014)	76 (100%)	35.5	27.6	19.7	52.7
Moszczyńska and cols. (2022)	23 (100%)	43.5	73.9	13	13.1
Li and cols. (2023)	28 (100%)	64.3	46.4	3.6	50
Zepeda and cols. (2023)*	9 (100%)	77.8	88.9	11.1	0

* The number of neoplastic causes in our cohort is higher than that in published studies, as we included only pediatric patients.

Abbreviations: LCH, Langerhans cell histiocytosis; GCT, germ cell tumor; ND, not described.

In our case series, germ cell tumor markers were negative both in serum and CSF in all patients. Notably, eight patients had a biopsy-confirmed diagnosis of pure germ cell tumors. These tumors may secrete hCG and AFP in serum and CSF through their syncytiotrophoblastic component (6). However, the secreted amounts are typically low, making these tumor markers generally undetectable. The value of hCG and AFP in the diagnosis of germ cell tumors has other pitfalls. First, the sensitivity and specificity of these markers vary depending on the anatomical location of the tumors, disease progression, and tumor subtypes, with markedly elevated concentrations in subtypes of germ cell tumors seen infrequently (18). Second, not all immunoassays have been validated for measurement of hCG and AFP in CSF (19). Third, the cutoff values for the normal range of these markers are not well established. One study attempting to determine the cutoff values providing the best sensitivity and specificity for both markers reported that the combination of CSF hCG ≥ 8.2 IU/L, serum hCG ≥ 2.5 IU/L, CSF AFP ≥ 3.8 ng/mL, and serum AFP ≥ 25 ng/mL yielded a total diagnostic sensitivity of 65.4% (20). Because of these limitations, new tumor markers for germ cell tumors are under investigation, with microRNAs showing promising results in recent publications (21).

Eight patients in the present study underwent transsphenoidal biopsy because they met the criteria for PST, as defined in the UK consensus (4). All of them had progressive PST with visual and/or pituitary involvement and negative first- and second-line investigations. In the ninth patient, the diagnosis was established by liver biopsy. The etiology of the PST remained unclear even after biopsy in four patients, who then required a second biopsy. Three of these patients had a pathological diagnosis of hypophysitis in the first biopsy, which is an extremely rare cause of PST in pediatric patients. Notably, patients with germ cell tumors can elicit an immune response against the tumor, leading to an inflammatory peritumoral reaction that can be misdiagnosed as hypophysitis (22). In these three cases, the final diagnosis after the second biopsy was of a germ cell tumor. Interestingly, one patient was first diagnosed with Langerhans cell histiocytosis by clinical and neuroimaging findings but, due to progressive enlargement of the pituitary stalk despite treatment, underwent a biopsy of the pituitary stalk, which was consistent with germ cell tumor.

Based on national statistics, germ cell tumors with pituitary stalk involvement are estimated to affect two patients annually in Chile (23). However, we observed an annual occurrence of four pediatric patients with PST due to germ cell tumors in the country during the study period. Thus, the frequency of patients with germ cell tumors doubled during the observed period. The reason for this increased frequency is unclear but may involve factors such as delayed diagnosis, growing interest among physicians in reporting infrequent manifestations of central nervous neoplasms, and a lack of specific studies for PST workup in some centers.

The main strengths of the present study are its status as the first report of pediatric patients with PST in Chile and Latin America, and its comprehensive clinical, endocrinological, radiological, and histological description of the cases. However, the study also has limitations, including its potential for having missed cases due to underreporting of non-neoplastic causes of PST in smaller centers. Still, this case series raises important awareness of PST, which is a rare condition. We analyzed the cases over a period of 3 years, and found a surprisingly higher frequency of PST among pediatric patients in Chile compared with published studies from other countries. It is crucial to monitor whether this trend continues in the future.

The management of patients with PST is complex and requires the collaboration of adult and pediatric neuroendocrinologists and experienced neurosurgeons, neuroradiologists, neuro-oncologists, neuro-ophthalmologists, and neuropathologists. These patients require long-term follow-up, as diagnostic tests for PST have some limitations. Thus, it is important to establish new markers and more precise diagnostic tools for promptly diagnosing PST and reducing unnecessary risks for our patients. The reasons for the increased frequency of PST in our cohort, particularly among girls, are still under investigation and remain to be elucidated.
